# Patients’ experiences of the use of point-of-care ultrasound in general practice – a cross-sectional study

**DOI:** 10.1186/s12875-021-01459-z

**Published:** 2021-06-18

**Authors:** Camilla Aakjær Andersen, John Brodersen, Torsten Rahbek Rudbæk, Martin Bach Jensen

**Affiliations:** 1 Center for General Practice, Aalborg University, Fyrkildevej 7, 13, 9220 Aalborg Øst, Denmark; 2 Research Unit for General Practice and Section of General Practice Department of Public Health, Faculty of Health Sciences, University of Copenhagen, Øster Farimagsgade 5, P. O. Box 2099, DK-1014 Copenhagen, Denmark; 3 Primary Health Care Research Unit, Region Zealand, Denmark; 4 Lægerne J.B. Winsløws Vej, J. B. Winsløws Vej 9a, 5000 Odense C, Denmark

**Keywords:** General practice, Family medicine, Primary care, Point-of-Care testing, Ultrasonography, Patient-reported outcome measures

## Abstract

**Background:**

The use of point-of-care ultrasonography (POCUS) performed by general practitioners (GPs) in primary care settings is increasing. Previous studies have focused on GP-reported outcomes and little is known about patients’ perspectives on the use of POCUS technology within the general practice consultation. The purpose of this study was to examine patients’ experiences with POCUS in general practice within the areas where GPs have indicated that POCUS affected aspects of the consultation.

**Methods:**

A questionnaire was developed using a mixed methods sequential design. Analytical themes from interviews with GPs were converted into items in a questionnaire by the research team. The questionnaire was then further developed in several rounds of pilot tests involving both patients and GPs. The final questionnaire was used in a cohort study conducted in 18 Danish office-based general practice clinics from January 2018 to August 2018. All patients examined with POCUS were asked to complete the questionnaire on tablets immediately after their consultation.

**Results:**

Out of 691 patients examined, 564 (81.6%) questionnaires were available for analysis. The patients reported that they were well informed about the purpose (98%) and the results (97%) of the POCUS examination; however, 29% reported that they were not informed about the difference between POCUS and an imaging-specialist’s ultrasound examination. Almost all patients (99%) reported that POCUS was integrated naturally into the consultation, and 45% reported that POCUS improved the doctor-patient relationship.

The majority of patients felt that they had been more thoroughly examined (92%) and taken more seriously (58%) when POCUS was part of the consultation. They felt POCUS gave them a better understanding of their health problem (82%), made them feel more secure (86%) and increased their trust in the physician’s assessment (65%). Moreover, the patients reported that POCUS use improved the level of service (95%) they experienced and the quality of care (94%) in general practice.

**Conclusion:**

We found that an examination including POCUS in general practice was a positive experience overall for the majority of patients. Future research should further explore reasons for patient confidence in POCUS and whether or not the reassuring value of POCUS is valid.

**Trial registration:**

ClinicalTrials.gov Identifier: NCT03416608

**Supplementary Information:**

The online version contains supplementary material available at 10.1186/s12875-021-01459-z.

## Background

Evaluating patients’ perspectives is paramount when offering health care services [1]. For patients, both access to health care and quality of care are likely to be priorities [2]. Access to tools and tests in primary care that would traditionally only be available at hospitals may improve diagnostic capability at the front line, and could be seen by patients as an improvement in the provision of health care service overall. There is a risk, however, that a reallocation of resources and priorities may lead to maldistribution of medical resources resulting in a poorer health care service [3].

General practitioners (GPs) are increasingly using point-of-care ultrasound (POCUS) in their examination of selected patients [4–6]. Patients may regard diagnostic POCUS as an improved service to ensure earlier and more correct diagnosis and treatment. However, evidence for such positive effects from POCUS largely originates from hospital settings while the evidence from a general practice setting is scant [7–10]. As the epidemiology, presentation and spectrum of symptoms, pathology and diseases differ between primary and secondary care, the performance of a test will inevitably differ between the two sectors [11]. Still, a growing number of publications describe the use of POCUS within family medicine or office-based general practice [10, 12]. There are few studies describing essential long-terms outcomes i.e. diagnostic test accuracy including overdiagnosis, mortality, morbidity and health economics. Very few studies have explored the potential intended benefits and the potential unintended harms of POCUS use in general practice from the patient’s perspective, or the patient’s reaction to the introduction of POCUS technology in general practice [13–17].

In emergency medicine, POCUS has been reported to increase patient satisfaction [18, 19], but patient satisfaction is complex, difficult to measure as a single concept and several confounders need to be considered [20]. In general practice, patients usually seek care for less acute conditions than in emergency medicine. The general practice consultation is characterised by an emphasis on communication, continuity of care and a strong doctor-patient relationship [1, 21]. Introducing a time-consuming diagnostic test, like POCUS, may disturb the structure of the consultation and give less room for dialogue and the patient’s agenda. Indeed, one study from general practice in Norway [13] found that 29% of patients felt that GPs were putting too much emphasis on technology in the consultation, and 19% found that the ultrasound examination disturbed the doctor-patient relationship. In other settings, POCUS has been found to increase the physician’s confidence and the patient’s satisfaction with the physician’s skills and abilities [19]. Whether this finding also applies to office-based general practice has not yet been investigated.

We have previously explored GPs’ perspectives on the use of POCUS in an interview study [22]. According to the GPs, patients appreciated the use of POCUS and the reassurance it provided for them. They felt POCUS use improved the doctor-patient relationship, increased patient confidence and respect for the GP. However, the GPs also expressed concerns about patients’ trust in the technology and in their own ability to communicate the limitations of POCUS use in general practice. These findings highlighted the need to assess patients’ perspectives on the use of POCUS in the general practice consultation.

In the present study, we aimed to explore patients’ experiences with POCUS use in general practice within the areas where GPs indicated in a previous study [22] that POCUS had affected aspects of the consultation. Specifically, we aimed to explore the POCUS-related information provided to the patient, the influence of POCUS on the consultation, patient reassurance and patient satisfaction.

## Methods

This cross-sectional study was developed using a mixed-method explorative sequential design [23]. Qualitative data collected in an interview study exploring the POCUS experiences of Danish GPs [22] was used to develop a quantitative patient questionnaire used in a cohort study measuring the use of POCUS in Danish general practice [24]. The protocol was prospectively registered in clinical trials (registration number: NCT03416608 10/01/2018) and reporting followed the STROBE checklist for cross-sectional studies.

### Setting

This study was conducted in Danish, office-based general practices, where GPs were already using POCUS [24]. Denmark has a public health care system where patients are listed with a GP for primary health care. GPs act as gatekeepers to other treatment options in primary care and to secondary healthcare through a referral system. Consultations are free of change for patients, as GPs are paid through tax-financed remuneration and fee-for service [25]. Only a few Danish GPs perform POCUS and they receive no fee for the service.

### Participants and recruitment

Twenty GPs working in 18 general practices were enrolled stepwise between January 15th 2018 and August 15th 2018 to account for seasonal variation. During a full month within the timeframe, each participating GP invited all patients, who had been examined with POCUS in the practice, to participated in this study. The general practice clinics were solo-practices and partnership-practices of different sizes and were located in both urban and rural areas. The participating GPs had different POCUS training and background characteristics which have been reported previously [24]. To be included in the study, patients had to be listed with the GP and be able to provide informed consent. In case of children, one or both parents had to provide informed consent depending on their custody arrangements.

### Data collection

There was no experimental intervention in this study as the GPs were already using POCUS in their examination of patients. The POCUS examinations performed reflected normal daily practice in the included clinics, and apart from project information and data collections, the study did not influence patient assessment and treatment. POCUS was used for both diagnostic and procure-related purposes. A variety of different organs were scanned including heart, lung, abdominal, gynaecological, obstetric and musculoskeletal examinations [24].

After the consultation, patients examined with POCUS were asked to complete a questionnaire using tablets provided by the project. The GPs accessed the online questionnaire on the tablets using a SurveyXact (Rambøll, Aarhus, Denmark) app, and inserted a unique de-identifier number for each patient before handing over the tablet to the patient. The patients completed the questionnaire in the waiting room of the clinic and data were transferred directly to the SurveyXact database. A paper edition of the questionnaire was available for patients who felt uncomfortable using a tablet. The GPs had no access to the completed patient questionnaires.

In a separate questionnaire, GPs registered data on the examined patients using the same de-identifier numbers. The main results of this registration have been reported previously [24]. For the present study, we included the GPs’ registration of their confidence in their main tentative diagnosis after using POCUS.

### Developing the questionnaire

**Fig. 1:**
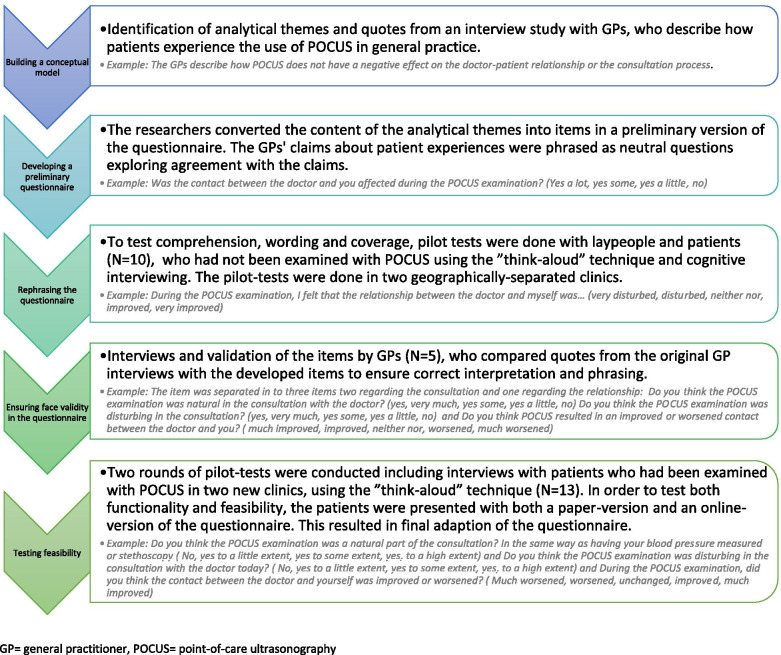
Developing the questionnaire. GP = general practitioner, POCUS = point-of-care ultrasonography

The items in the patient questionnaire were developed using mixed method integration. A preceding interview study with 13 Danish GPs who used POCUS in their daily practice [22] had identified the following themes regarding the patient experience: (1) Information given to the patient; POCUS influence on (2) the consultation, (3) patient reassurance, and (4) patient satisfaction. The research group converted the analytical text and quotes within each domain into items in a preliminary version of the questionnaire, which was then tested and further developed by involving both patients and GPs as elaborated in Fig. 1. The development of the GP questionnaire has been reported previously [24].

### Outcome measures

The patients were asked to provide the following background characteristics: age, gender, current employment, level of education and whether they had been examined with ultrasonography previously.

Furthermore, they were asked if they had received information about: (1) the purpose of the examination; (2) the result of the examination, and (3) information about the difference between a specialist’s ultrasound examination and a GP’s ultrasound examination.

Regarding the influence of POCUS on the consultation, patients were asked if they felt POCUS was: (1) a natural part of the consultation or (2) disruptive to the consultation process. The patients were also asked: (3) whether POCUS had affected the doctor-patient relationship and (4) whether they believed POCUS had made a difference to the treatment they received.

Dimensions of patient reassurance were explored by asking the patients if the use of POCUS had: (1) made them feel more or less thoroughly examined; (2) given them a better or poorer understanding of their health problem; (3) made them feel more or less secure; (4) increased or decreased their confidence in the GP’s assessment of their health problem, and (5) made them feel more or less taken seriously.

Dimensions of patient satisfaction were assessed by asking the patients if they felt the use of POCUS had increased or decreased: (1) the level of service, or (2) the overall quality of care. Patients were also asked to: (3) evaluate their overall experience with POCUS as either positive or negative, and to (4) declare if they were likely to recommend POCUS to other patients with the same health problem. The full patient questionnaire is available in Additional file 1.

In the GP questionnaire, the GP’s confidence in their main tentative diagnosis for the patient was registered after POCUS as either highly increased, increased, unchanged, decreased, or highly decreased confidence.

### Sample size

This was a first descriptive study, hence, no specific sample size calculation was made. Based on a previous questionnaire study from 2016 [4], we estimated that around 75 Danish GPs were using POCUS at the time of the study. We believed that we would be able to recruit 20 of these to participate in the study. Based on the interview study [22], we estimated that GPs would use POCUS 2–3 times a day, and assuming a participation rate of 80%, we expected to include a total of 640–960 patients.

### Statistics

Data were collected using single-choice items with categorical response categories collected on nominal or assumed ordinal scales. Therefore, the results were reported using frequencies. Associations between the GPs’ reported *confidence in the tentative diagnosis* on an ordinal scale from highly decreased confidence to highly increased confidence, and the dimensions of *patient’s reassurance* was tested using Goodman and Kruskal’s gamma. Fischers exact test was used to test if the found frequencies statistically differed from the expected frequencies. A test probability of less than 0.05 was considered statistically significant.

## Results

**Fig. 2:**
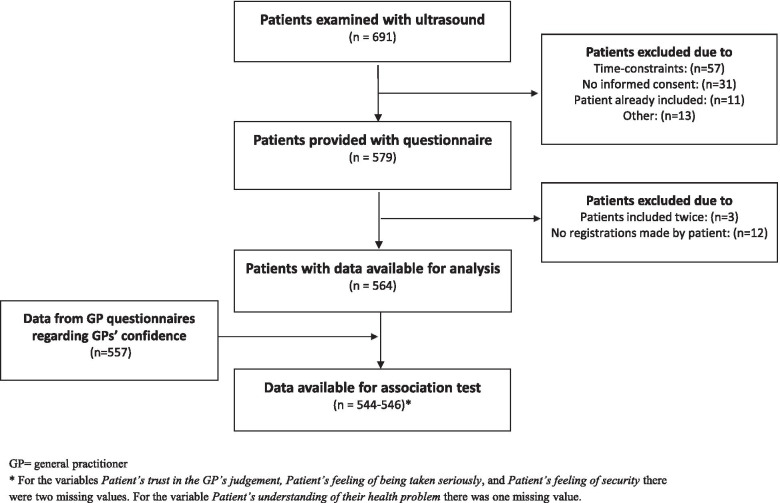
Patients included in the study. GP = general practitioner. * For the variables Patient’s trust in the GP’s judgement, Patient’s feeling of being taken seriously, and Patient’s feeling of security there were two missing values. For the variable Patient’s understanding of their health problem there was one missing value

A total of 691 patients were examined with POCUS corresponding to an individual average daily use per GP between 0.6 and 3.9 ultrasound examinations. Patient questionnaires from 564 patients were available for analysis (Fig. 2). Background characteristics of participating patients are presented in Table 1.

**Table 1:** Patient characteristics

**Patient characteristics***(N* = *564)*	**N (%)**
**Age**^**a**^	
< 20 years	13 (2)
20–39 years	164 (29)
40–59 years	199 (35)
60–79 years	157 (28)
> 79 years	29 (5)
**Gender**^**a**^	
Male	186 (33)
Female	376 (67)
**Employment**	
Currently working	308 (55)
Unemployed	15 (3)
Currently a student	44 (8)
Retired	160 (28)
Other	33 (6)
**Level of education after primary school**	
Short specialized training^b^	6 (1)
Trade/technical/vocational training^b^	126 (22)
Short education (corresponding to an Associate’s degree)	67 (12)
Medium education (corresponding to a Bachelor’s degree)	159 ( 28)
Longer education (corresponding to a Master’s degree)	61 (11)
Other education	68 (12)
Currently a student	25 ( 4)
Do not know	39 (7)
**Place of residence**	
Capital Region of Denmark	145 (26)
Region Zealand	25 (4)
Region of Southern Denmark	190 (34)
Central Denmark Region	99 (18)
North Denmark Region	105 (19)

*POCUS* Point-of-care ultrasonography

^a^In comparison missing patients had a mean age of 46 years and were 50% male

^b^ No high-school requirement

### Information given to the patient

Ninety-eight percent of patient participants reported that they felt very informed (*N* = 488) or informed to some extent (*N* = 65) about the purpose of the POCUS examination, whereas fewer than 2% felt informed to a lesser extent (*N* = 9) or not informed (*N* = 1). Ninety-seven percent reported that they had been informed about the result of the POCUS examination (very informed: *N* = 456; informed to some extent: *N* = 81 or to a lesser extent: *N* = 20), and only 1% reported that they were not informed about the results of the ultrasound examination (*N* = 5).

Regarding information about the difference between a POCUS examination performed by a GP in primary care and a specialist’s examination in the secondary sector, patients reported that they had been: very informed: 28% (*N* = 158); informed to some extent: 27% (*N* = 150); informed to a lesser extent:16% (*N* = 88), or not informed about this difference: 29% (*N* = 165).

### POCUS influence on the consultation

Ninety-nine percent of patients reported that POCUS was integrated as a natural part of the GP consultation (to a high extent: *N* = 471; to some extent: *N* = 75; to a lesser extent: *N* = 13). Ninety-six percent reported that POCUS was not disruptive in the consultation, while the remaining 4% said that POCUS was disruptive (to a high extent: *N* = 7; to some extent: *N* = 4, or to a lesser extent: *N* = 4).

Fifty-five percent (*N* = 310) reported that POCUS did not affect the doctor-patient relationship, whereas 45% reported that they felt the doctor-patient relationship was very much improved (*N* = 105) or improved (*N* = 146) by POCUS. Only one patient reported that POCUS made the relationship worse.

### POCUS influence on patients’ sense of reassurance

**Fig. 3:**
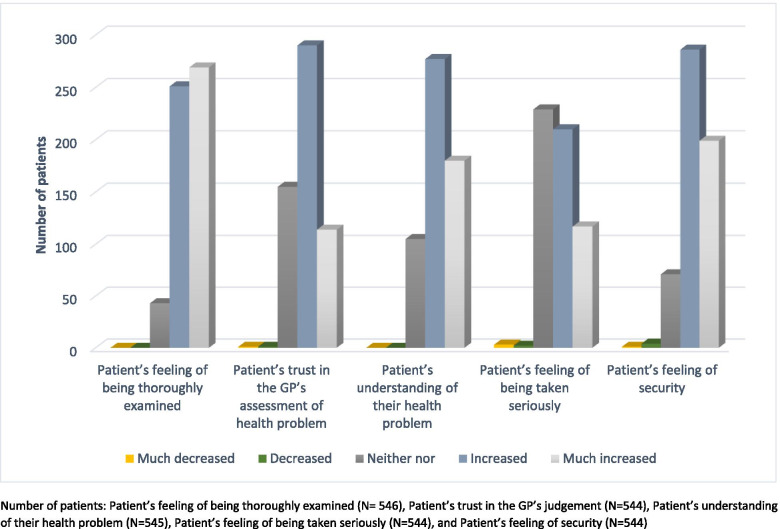
Dimensions of patient reassurance measured in the questionnaire. Number of patients: Patient’s feeling of being thoroughly examined (*N* = 546), Patient’s trust in the GP’s judgement (*N* = 544), Patient’s understanding of their health problem (*N* = 545), Patient’s feeling of being taken seriously (*N* = 544), and Patient’s feeling of security (*N* = 544)

As illustrated in Fig. 3, 92% of patients reported that they felt much more thoroughly (*N* = 269) or more thoroughly (*N* = 251) examined after POCUS, while the remaining patients reported that POCUS had no influence in that regard (*N* = 43). Notably, no patients reported that POCUS made them feel less thoroughly examined.

Eighty-one percent reported that POCUS had provided them with a much better (*N* = 180) or better (*N* = 277) understanding of their health problem, while 19% reported that POCUS had not changed their understanding (*N* = 105). No patients reported that POCUS had given them a poorer understanding.

Eighty-six percent reported that POCUS had made them feel much more secure (*N* = 199) or more secure (*N* = 286), while 13% (*N* = 71) felt POCUS had made no change in that regard and 1% felt POCUS had made them feel much less secure (*N* = 1) or less secure (*N* = 4).

Sixty-five percent reported that their trust in the GP’s assessment of their health problem was much increased (*N* = 114) or increased (*N* = 290) after POCUS; 27% (*N* = 155) reported that their trust had not changed, and fewer than 1% reported that their trust had decreased (*N* = 2).

Fifty-eight percent reported that the use of POCUS had made them feel that they had been taken much more seriously (*N* = 117) or more seriously (*N* = 210); 41% reported that POCUS had made no change in that regard, while 1% reported that they felt they had been taken much less seriously (*N* = 3) or less seriously (*N* = 2).

### Improved care

Eighty percent of patients reported that they believed POCUS had a very large influence (*N* = 129) or a large influence (*N* = 320) on the care that they received at the GP’s office, and 5% reported that POCUS had very little influence (*N* = 2) or little influence (*N* = 24) on the care that they had received. Fifteen percent reported no difference (*N* = 87).

In more general terms, 95% of patients reported that POCUS very much improved (*N* = 287) or improved (*N* = 253) the service at the GP’s office, while 4% reported no difference*.* Ninety-four percent reported that POCUS very much improved (*N* = 250) or improved (*N* = 278) the quality of care in general practice while 4% reported no difference*.* Notably, no patients found that POCUS decreased service or quality of care in general practice.

### Overall patient experience

Ninety-six percent of patients reported that they had a very positive (*N* = 334) or positive (*N* = 220) experience of being examined using POCUS at the GP’s office. The remaining eight patients reported that the experience had been neither positive nor negative. Hence, no patients reported having a negative overall experience.

Ninety-two percent reported that they would most likely (*N* = 351) or likely (*N* = 169) recommend the POCUS examination to others; 7% reported that they would neither recommend nor discourage the examination, and one patient reported that it was most unlikely that they would recommend the examination to others.

### Association between patient reassurance and GP confidence

**Fig. 4:**
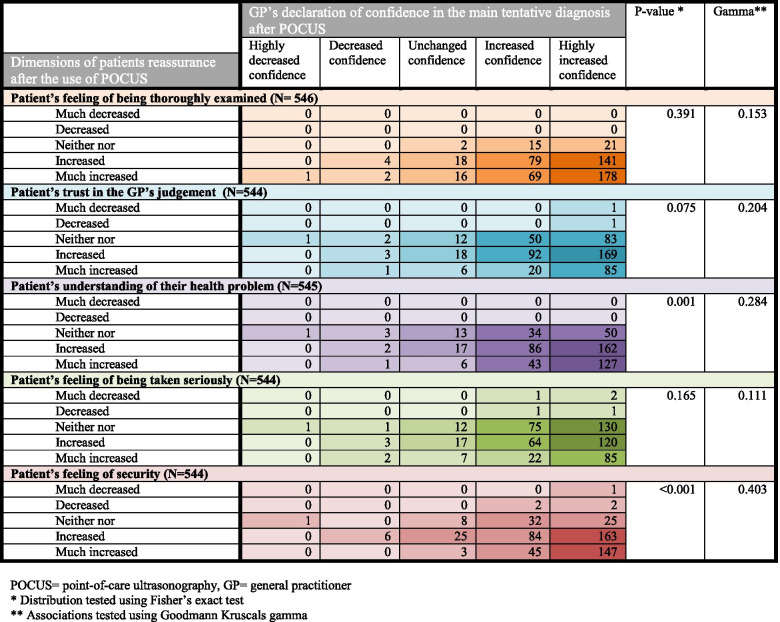
Associations between patient reassurance and general practitioners’ confidence in diagnosis. POCUS = point-of-care ultrasonography, GP = general practitioner. * Distribution tested using Fisher’s exact test. ** Associations tested using Goodmann Kruscals gamma

As illustrated in Fig. 4, the distribution of data was unidirectional. However, we found no strong associations between the GPs’ confidence in the main diagnosis and the dimensions of patient reassurance. A relationship beyond chance was found between the GPs’ confidence in the main diagnosis after POCUS and the patient’s declaration of increased understanding of their health problem, and the patient’s sense of security.

## Discussion

### Summary of main findings

In this study, we found that most patients felt they were well informed about the purpose and the results of the POCUS examinations. However, only half of the patients felt very informed or informed to some extent about the difference between a GP POCUS and an imaging-specialist’s ultrasound examination. Almost all (99%) patients reported that POCUS was integrated naturally into the GP consultation and 45% reported that POCUS may even have improved the doctor-patient relationship.

POCUS use had a large influence on patient reassurance: 92% felt more thoroughly examined, 85% felt they had been taken more seriously, and 86% felt more secure after being examined using POCUS. Eighty-eight percent reported an increased understanding of their health problem and 65% had an increased trust in the GP’s assessment of their health problem after the use of POCUS. Although we found that the majority of GPs reported an increased confidence in their diagnosis after POCUS, no strong association was found between the GPs’ increased confidence and the patients’ sense of reassurance.

Finally, we found that the vast majority of patients said that POCUS use improved their experience of the level of service and quality of care in general practice, and no patients reported having a negative experience with the use of POCUS in general practice.

### Strengths and limitations

The questionnaire was developed based on extensive preceding qualitative work. However, the items originated from interviews with GPs and not patients. Hence, it is possible that interviews with patients preceding this study would have revealed aspects of the patient experience that we did not include. Nevertheless, all dimensions of the items in the questionnaire were pilot-tested with patients giving some assurance that they were relevant to patient experience.

Although, we had a 97% participation rate, we had expected to include more patients. The participating GPs reported that the study registrations were time consuming and, therefore, fewer patients were examined with POCUS during the study period. A larger sample may have provided more diversity in the results.

Generalisability may have been compromised as the GPs performing POCUS in this study are likely to constitute a selected sub-group of GPs with a special interest in ultrasonography. These GPs choose to use POCUS in their daily work without receiving a fee or remuneration for the extra service or their time. It is possible that the GPs’ enthusiasm for the technology affected the consultations and created a more positive atmosphere, which may have influenced patients’ experiences. Hence, it is unknown if similar results would be found in a larger or more diverse group of GPs.

To avoid recall bias, patients completed the questionnaires in the clinic’s waiting room immediately after their consultation, but this may have affected their responses. The high response rate could reflect the inability of some patients to say ‘no’ to participation, as the questionnaire was presented to them by the GP. Hence, we cannot reject some potential response bias where patients’ answers are in favour of their GPs.

This was a first descriptive study examining patient experiences with the use of POCUS in general practice, and as such we did not adjust for possible confounders. However, the distribution of data was strongly unidirectional. The lack of a comparator in our study design makes us unable to determine whether our findings are, in fact, a result of the use of POCUS or a more general expression of loyalty, trust and satisfaction with the GP. However, the phrasing of the questions was specifically designed to elicit the impact of POCUS.

### Findings in context

Despite a growing number of publications describing the use of POCUS in general practice, little attention has been given to patients’ perspectives on the use of the technology [12]. Previous studies have explored patient experiences with POCUS in general practice using questionnaires administered immediately after the consultation [16], after 15 to 30 days [15], and after three [13] or four [17] months following the examination. Although using various designs and items to measure patients’ experiences, all studies reported that POCUS aligned well with patient preferences.

Accordingly, we found an overall positive experience with the GPs’ use of POCUS assessed immediately after the consultation. Three previous studies from general practice found that patients preferred having the examination performed at the GP’s office rather than going to the hospital [15–17]. Using discrete-choice methodology, one of the studies from a rural general practice [16] even found that patients were willing to trade off diagnostic accuracy to have the examination performed locally at the GP’s office. Hence, the high patient satisfaction reported in the present study may be explained merely by the availability of the test and patient expectations that the GP will take an active diagnostic approach in the consultation [26–29]. However, a POCUS examination is not a replacement for the traditional comprehensive ultrasound examination performed by an imaging-specialist [30, 31]. POCUS examinations are typically restricted to ruling in or ruling out a specific condition e.g. the presence or absence of a gallbladder stone, without exploring the surrounding areas. The premise for POCUS is that it is an abbreviated procedure, acceptable for the purpose. POCUS is superior to traditional ultrasound examinations in terms of accessibility, speed and availability, but it is inferior in terms of range and its ability to rule out disease [30]. Awareness of the limitations and communicating the differences between the two examinations is important to avoid false expectations about what the examinations can provide. We found that not all patients reported having received such information. This might confirm the findings from the preceding qualitative study [22], where GPs reported that, despite informing patients about the limitations of POCUS, they felt unsure about whether patients understood the differences between a specialist’s and a generalist’s examination. A perception gap between the information provided and the patients’ understanding of the information has been found regarding other tests too [32].

The GPs in our study all used POCUS on a weekly basis. However, their training and usage varied a lot, resulting in different competence levels [33]. Our study did not compare how this might have affected the patients’ perceptions of trust and confidence in the examination. Another possible explanation for high patient satisfaction with POCUS use is the reliance on diagnostic tests. Patients have been found to appreciate the use of point-of-care tests in general practice [27] and to put a lot of emphasis on diagnostic tests [26, 34, 35]without necessarily understanding their limitations, pitfalls and the potential unintended harms [34]. Studies have found that for some diseases reliance on the traditional physical examination of patients is questionable [36, 37]and patients, as well as doctors, may be aware of this. The mere availability of a diagnostic test may lead to wish-fulfilling medicine, where diagnostic examinations are performed upon patient request to meet patient expectations or to provide reassurance [38]. GPs undertaking POCUS must be aware of this risk and communicate their medical reasoning to patients. However, providing reassurance for patients is both important and common in general practice [39] and GPs have been found to use diagnostic tests to reassure themselves and patients [35, 40]. Measuring dimensions of reassurance immediately after the consultation, we found that patients felt an increased trust in their GP and a sense of security. However, studies have suggested that the immediate reassuring value of pregnancy-related ultrasound is not long-lasting [34, 41]and evidence does not support long-term reassurance by diagnostic tests [42, 43].

The diagnostic accuracy of a test and the pre-test probability of a condition have to be taken into account before GPs and patients can actually be assured that a diagnostic test (including POCUS) can, with reasonable certainty, rule in or rule out a condition [45]. GPs performing POCUS must be aware of this and the potential false reassurance that having a POCUS examination may provide for patients and themselves. Likewise, GPs must consider the risk of overdiagnosis, overdetection and possibly overtreatment following the introduction of an additional test [12, 45, 46]. Hence, GPs must continue to practice generalist medicine up to a certain level before handing the patient over to secondary care, informing patients about the limitations and risks of point-of-care examinations and referring patients to imaging specialists in case of doubt.

### Implications

The increasing pressure on general practice in terms of more elderly and multi-morbid patients and more treatments transferred to primary care calls for faster and more precise diagnoses at the GP’s office. POCUS may be a valuable tool in this respect, but prudence must be exercised to ensure the quality of the examinations performed by GPs and the correct allocation of health care resources. The availability of high-resolution, affordable and portable ultrasound devices, together with the introduction of POCUS training in medical schools [47, 48] and residency programmes [49], makes it plausible that POCUS will be more commonly used in general practice in the future. This study suggests that patients appreciate POCUS use in general practice, however, a thorough evaluation of POCUS use should include an evaluation of experience of care, population prognoses and health and cost of care [50]. Although recent studies describing the use of focused POCUS in general practice have reported promising results [12], more high-quality research is needed to evaluate the diagnostic precision and impact of POCUS use in general practice including the patients’ prognoses and the impact on the health care sector as a whole.

## Conclusion

We found that being examined with POCUS by GPs in general practice was a positive experience overall for the majority of patients. They felt that POCUS was integrated as a natural part of the consultation and most patients felt reassured after the POCUS examination. More than nine out of ten patients reported that POCUS increased the perception of level of service and quality of care in general practice. Generally, patients reported that they were well informed about why they needed the POCUS examination and the results, but almost half of patients felt ill-informed about the differences between POCUS in general practice and an imaging specialist’s ultrasound examination.

## Supplementary Information

**Additional file 1**

## Data Availability

The anonymised questionnaires and datasets are available and stored at Center for General practice at Aalborg University, Denmark according to regulations by the Danish Data Protection Agency. Anonymised data are available on request by contacting the corresponding author.

## Abbreviations

GP: General Practitioner
POCUS: Point-of-care ultrasonography
