# Supplementary material for: Patients’ experiences of the use of point-of-care ultrasound in general practice – a cross-sectional study
Source: BMC Fam Pract. 2021 Jun 18;22:116. doi: 10.1186/s12875-021-01459-z (PMC8214303; doi:10.1186/s12875-021-01459-z)
Supplement: Supplementary file 1 — Additional file 1 [file 12875_2021_1459_MOESM1_ESM.docx]

Additional file 1.

Questionnaire given to patients in general practice after the consultation, where they were examined with point-of-care ultrasonography

**Manuscript title:** Patients’ Experiences of the Use of Point-of-Care Ultrasound in General Practice – a Cross-Sectional Study.

**Manuscript authors:** Camilla Aakjær Andersen, PhD, MD; John Brodersen, Professor, PhD, MD; Torsten Rahbek Rudbæk, MD; Martin Bach Jensen, Professor, PhD, MD

# Patient questionnaire

| 1. The first questions concern the information you received about the ultrasound examination performed by the physician today. | | | | |
| --- | --- | --- | --- | --- |
|  | No | Yes, to a little extend | Yes, to some extend | Yes, to a high extend |
| Were you informed about the purpose of the ultrasound examination? | 🞏 | 🞏 | 🞏 | 🞏 |
| Were you informed about the difference between a specialist ultrasound and a GP ultrasound? | 🞏 | 🞏 | 🞏 | 🞏 |
| Were you informed about the result of the ultrasound examination? | 🞏 | 🞏 | 🞏 | 🞏 |

| 2. The following questions concern your experience during the ultrasound examination performed by the physician today. | | | | |
| --- | --- | --- | --- | --- |
|  | No | Yes, to a little extend | Yes, to some extend | Yes, to a high extend |
| Do you think the POCUS examination was a natural part of the consultation? *(In the same way as having your blood pressure measured or stethoscopy)* | 🞏 | 🞏 | 🞏 | 🞏 |
| Do you think the POCUS examination was disturbing in the consultation with the doctor today? | 🞏 | 🞏 | 🞏 | 🞏 |

| During the POCUS examination, did you think the contact between the doctor and yourself was improved or worsened? | Much worsened  🞏 | Worsened  🞏 | Unchanged  🞏 | Improved 🞏 | Much improved  🞏 |
| --- | --- | --- | --- | --- | --- |

| 3. The following questions concern your experience after having received an ultrasound examination performed by your physician today. | | | | | |
| --- | --- | --- | --- | --- | --- |
| Do you think the ultrasound examination has given you a better or poorer understanding of your health problem? | Much poorer understanding  🞏 | Poorer understanding  🞏 | Neither nor  🞏 | Better understanding  🞏 | Much better understanding  🞏 |
| Do you feel more thoroughly or less thoroughly examined after the ultrasound examination? | Much less thoroughly  🞏 | Less thoroughly  🞏 | Neither nor  🞏 | More thoroughly  🞏 | Much more thoroughly  🞏 |
| After the ultrasound examination, do you fell more or less taken seriously. | Much less taken seriously  🞏 | Less taken seriously  🞏 | Neither nor  🞏 | More taken seriously  🞏 | Much more taken seriously  🞏 |
| After the ultrasound examination, do you feel more or less secure? | Much less secure  🞏 | Less secure  🞏 | Neither nor  🞏 | More secure  🞏 | Much more secure  🞏 |
| Do you think the ultrasound examination had a large or small impact on the treatment you received? | Very small impact  🞏 | Small impact  🞏 | Neither nor  🞏 | Large impact  🞏 | Very large impact  🞏 |
| After the ultrasound examination, has your confidence in the GPs assessment of their health problem increased or decreased? | Much decreased  🞏 | Decreased  🞏 | Neither nor  🞏 | Increased  🞏 | Much increased  🞏 |

| 4. The following questions concern you overall experience with the ultrasound examination performed by your physician today | | | | | |
| --- | --- | --- | --- | --- | --- |
| Overall, do you think the ultrasound examination makes the level of service at the GP’s office increase or decrease? | Much decreased service  🞏 | Decreased service  🞏 | Neither nor  🞏 | Improved service  🞏 | Much improved service  🞏 |
| Overall, do you think the ultrasound examination makes the quality in care at the GP’s office increase or decrease? | Much decreased quality  🞏 | Decreased quality  🞏 | Neither nor  🞏 | Better quality  🞏 | Much better quality  🞏 |
| How was your overall experience with the ultrasound examination performed by the physician today? | Very negativ  🞏 | Negativ  🞏 | Neither nor  🞏 | Positiv  🞏 | Very positiv  🞏 |
| How likely are you to recommend the ultrasound examination to other patients having the same health problem and the same physician? | Very unlikely  🞏 | Unlikely  🞏 | Neither nor  🞏 | Likely  🞏 | Very likely  🞏 |

5. The last questions are about you

Are You…

(1) ❑ Male

(2) ❑ Female

Please insert Your year of birth

_____

Please choose Your employment

(1) ❑ currently working

(2) ❑ currently a student

(3) ❑ currently unemployed

(5) ❑ retired

(6) ❑ other

**Please insert Your level of basic school education**

(1) ❑ 7th-9th grade

(2) ❑ 10th grade

(3) ❑ High school

(4) ❑ Other

(5) ❑ Do not know

Please insert Your level of education

(1) ❑ Short specialized education

(2) ❑ Longer specialized education

(3) ❑ Short further education after high school

(4) ❑ Medium further education after high school

(5) ❑ Long further education after high school

(6) ❑ Other education

(7) ❑ Currently a student

(8) ❑ Do not know

**Have You been examined with ultrasonography before?**

(1) ❑ No

(2) ❑ Yes, here in general practice

(3) ❑ Yes, at another primary care clinic

(4) ❑ Yes, at a specialist clinic (e.g. a gynecology clinic)

(5) ❑ Yes, at the hospital

(6) ❑ Do not know
